# Comparative Analysis of Flexural and Compressive Strengths of Bioactive Alkasite Compared to Other Ion-Releasing Restorative Materials

**DOI:** 10.3390/biomimetics10110751

**Published:** 2025-11-07

**Authors:** Hanin E. Yeslam, Fatin A. Hasanain

**Affiliations:** 1 Department of Restorative Dentistry, Faculty of Dentistry, King Abdulaziz University, Jeddah 21589, Saudi Arabia; fhasanain@kau.edu.sa; 2 Advanced Technology Dental Research Laboratory, Faculty of Dentistry, King Abdulaziz University, Jeddah 21589, Saudi Arabia

**Keywords:** composite resin, glass ionomer cements, alkasites, restorative dentistry, dental materials, bioactive materials, biomimetics, fluorides

## Abstract

Background: Ion-releasing and bioactive restorative materials are an integral part of restorative dentistry, especially in light of minimally invasive and esthetic intervention strategies. Their strength and mechanical properties directly influence their durability and indicated use. Methods: This study aimed to comparatively analyze the compressive strengths, flexural strengths, and flexural moduli of bioactive Alkasite (Cention N) and other ion-releasing restorative materials, specifically a resin-modified glass ionomer (RMGIC, Fuji II LC) and a compomer (Dyract XP). Cylindrical and bar-shaped specimens were fabricated from each material (*n* = 6 per material and conducted test) and subjected to mechanical strength testing (compressive and flexural strength) using a 2 kN cell universal testing machine (Instron 5944) with a crosshead speed of 0.5 mm/min. Statistical analysis, using one-way ANOVA and Tukey’s HSD post hoc tests, was conducted. Results: The results revealed significant differences in mechanical properties between the tested materials. Dyract XP showed the greatest compressive and flexural strengths (170.79 ± 23.59 MPa and 114.09 ± 30.78 MPa) (*p* < 0.01). Fuji II LC had a significantly greater flexural modulus (10.21 ± 4.46 GPa) than Dyract XP. Conclusions: The findings indicated that the investigated compomer could produce stronger restorations than the investigated alkasite and RMGIC, which would make them preferred for posterior teeth restoration. However, the alkasite Cention N might still be a good option for the treatment of carious lesions in areas with less occlusal stress.

## 1. Introduction

Dental caries is a persistent, multifactorial, non-communicable infectious disease influenced by oral biofilm and diet, affecting patients from children to the elderly with high prevalence [1]. This has led to a paradigm shift in managing these lesions, fostering the development of new restorative materials and techniques. The goal of minimal intervention dentistry using the biomimetic approach for treating carious lesions is to detect them early, promote remineralization, and manage active lesions in enamel or dentin through both surgical and non-surgical methods, utilizing materials that imitate natural tissues [2,3,4,5]. Promotion of remineralization and prevention of further caries lesion development can be achieved using bioactive restorative materials and topical traditional intervention with or without the adjunctive use of lasers at sub-ablative irradiation energy levels [6]. Clinical choices for materials are largely driven by their mechanical and biological properties, especially for high-caries-risk patients [7]. The compressive and flexural strengths of dental restoratives are directly related to the resultant restoration’s resistance to intraoral functional and parafunctional occlusal forces [8]. Unfortunately, most strong direct restorative materials lack antibacterial and remineralizing properties while risking failure and the development of secondary caries [9].

Recognizing the shortcomings of conventional dental materials has led to the search for options that provide extra benefits for dental health [10]. This has resulted in ion-releasing restorative polymeric materials that actively participate in the oral environment [10,11,12,13,14,15]. Some of these materials can mimic physiological conditions and integrate with dental tissues, reducing acid-induced dissolution of enamel and dentin [16]. These are often referred to as bioactive materials, although the definition has recently been refined [17,18]. Current research focuses on creating restorative materials with remineralizing, antibacterial, and self-healing properties, designed to repair hard tissue defects while providing these benefits. However, ion-releasing materials have drawbacks such as poor hydrolytic stability, low flexural strength, and toughness [14,19,20,21,22]. Reactive fillers may suffer a decline in fluoride release and decrease the mechanical properties of these materials, potentially compromising the durability of fabricated restorations [23].

The first ion-releasing restorative materials (IRRs) were conventional glass ionomer cements (GICs), consisting of fluoroaluminosilicate powder, a liquid polymer or copolymer of carboxylic acid, water, and tartaric acid [24]. They offer beneficial fluoride release and recharge, which aids in preventing demineralization and promoting remineralization, chemical bond formation with natural tooth structure, and antibacterial effects [25]. However, their tendency to wear and fracture prompted the development of resin-modified glass ionomer cements (RMGICs) with improved mechanical properties [26]. RMGICs are dual-cure materials that blend GIC components with methacrylate monomers and initiators, enabling longer working time, faster setting, and better esthetic and mechanical properties than conventional GICs [27,28]. They can induce marginal remineralization, but their strength, wear resistance, and esthetics still fall short compared to resin composite restorations [27,28]. These limitations have resulted in their reduced effectiveness as final restorations. Compomers, on the other hand, are hybrid polyacid-modified resin materials that combine resin composites with GIC, providing a light-cured material with enhanced mechanical and esthetic properties while releasing fluoride for caries prevention [29,30,31,32]. During maturation, their hydrophilic components attract a minute amount of moisture to facilitate an acid–base reaction and release clinically beneficial fluoride [33].

Recently, the alkasite resin material Cention N (Ivoclar Vivadent, Schaan, Liechtenstein) has been developed by incorporating alkaline calcium–fluor–silicate glass fillers and demonstrating superior mechanical properties [25,34,35,36]. The material can release fluoride, calcium, and hydroxide ions, remineralize tooth structure, and neutralize acidic pH caused by cariogenic bacteria [35]. It comprises a powder and liquid that begin to polymerize when mixed, thanks to a copper salt-based initiator system [37,38,39]. Additionally, it can be optionally light-cured because it contains an Ivocerin-based photoinitiator and acyl phosphine oxide [30]. Both light-cured and self-cured specimens of the Cention N material demonstrated equivalent strength values in a previous study [40]. This combination of properties and curing methods increased its appeal in clinical practice while improving the restoration’s durability [41]. Its reportedly higher strength compared to GICs, RMGICs, and resin-based composite and the limited related literature warrant its further evaluation as a definitive restorative material [30,42].

While some studies have examined the mechanical properties of alkasite materials, not much exists comparing them to newer formulations of ion-releasing materials. Given the importance of the dental restorative material’s mechanical properties for its durable performance intraorally, more research is needed to compare the mechanical properties of these materials. This comparison could improve clinicians’ evidence-based decision-making ability in daily clinical practice. This study evaluated three tooth-colored ion-releasing materials: an alkasite (Cention N, Ivoclar Vivadent, Schaan, Liechtenstein), a compomer (Dyract XP, Dentsply Sirona, Konstanz, Germany), and a resin-modified glass ionomer (Fuji II LC, GC Corporation, Tokyo, Japan). The goal was to assess their compressive strength and flexural behaviour and to determine their suitability as final, restorative options compared to traditional caries-preventive materials. The null hypothesis posits no differences in compressive strength, flexural strength, or flexural modulus between the alkasite (Cention N), resin-modified GIC (Fuji II LC), and the compomer (Dyract XP).

## 2. Materials and Methods

The composition of the three tooth-colored, ion-releasing restorative materials under investigation: Cention N (Ivoclar Vivadent, Schaan, Liechtenstein), Dyract XP (Dentsply Sirona, Konstanz, Germany), and Fuji II LC (GC Corporation, Leuven, Belgium), as supplied by the manufacturers, is detailed in Table 1.

### 2.1. Compressive Strength Specimen Preparation and Testing

**Figure 1:**
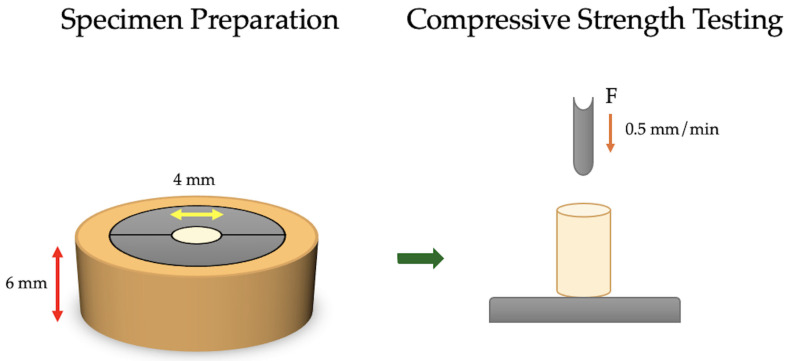
Compressive test specimen preparation and testing apparatus.

Custom stainless-steel molds were used to create cylindrical specimens (*n* = 6 per material), following ISO standard 9917-1:2025 (4 mm × 6 mm) [43]. The molds consisted of two half-circle sections, allowing for the easy removal of specimens from the cylindrical space without accidentally damaging them. An outer ring securely held the mold parts together during material packing and polymerization. The molds were cleaned with an alcohol swab between specimens, and air-dried to prevent cross-contamination that could interfere with the setting reaction and increase variability between specimens. The custom mold and testing apparatus are demonstrated in Figure 1.

All ion-releasing restorative materials (IRRs) were carefully prepared at room temperature following their respective manufacturers’ instructions. For Fuji II LC, capsules were first activated using a GIC applicator before being mixed in an amalgamator (Silamat S3, Ivoclar Vivadent Inc., Schaan, Liechtenstein) for 10 s. The capsules were then placed in the applicator to inject the Fuji II LC RMGIC into the molds. Dyract XP, a paste contained in a mini syringe, was injected into the molds using its designated applicator gun. Both Dyract XP and Fuji II LC pastes were injected until they completely filled the mold in one increment to reduce the risk of void formation. Cention N powder and liquid (1 scoop of powder with 1 drop of liquid) were mixed on a glass slab for 45 to 60 s. The resulting paste was then condensed in a single increment into the mold to prevent inadvertent void formation between increments. All materials were injected into the molds in a single increment. All specimens from each material were produced by a single operator to ensure consistency and reduce variability.

Two clear glass slides with a thickness of 1 mm on both the top and bottom surfaces were used to ensure uniform and straight specimen surfaces with a standardized distance from the light-cure tip. Then, specimens were light cured using a mono-wave LED light-curing unit (Elipar, 3M ESPE, St. Paul, MN, USA). The light cure unit’s irradiance of 1200 mW/cm^2^ was confirmed using a radiometer beforehand, to ensure consistency. The light cure was applied perpendicularly to both the top and bottom sides for 20 s per side. Afterwards, the samples were taken out of the molds by disassembling the mold parts and excess. All specimens were visually inspected, and any defective specimens were discarded and replaced with new ones. All specimens were stored in distilled water-filled dark containers at room temperature (25 °C) for 24 h before testing.

The compressive strength test was performed by loading the specimens till fracture in the 2 kN universal testing machine (Instron 5944, Instron Corp, Norwood, MA, USA) with a crosshead speed of 0.5 mm/min. A 3-mm round tip was used in the machine to apply the load on each specimen. The highest recorded load value at the point of fracture was registered, and the compressive strength was calculated using the following formula:

σc = 4P/(πD^2^)

where σc is compressive strength in (MPa); P is the maximum applied load in Newtons (N); and D is the diameter of the cylindrical specimens in millimeters (mm).

### 2.2. Flexural Properties’ Specimen Preparation and Testing

**Figure 2:**
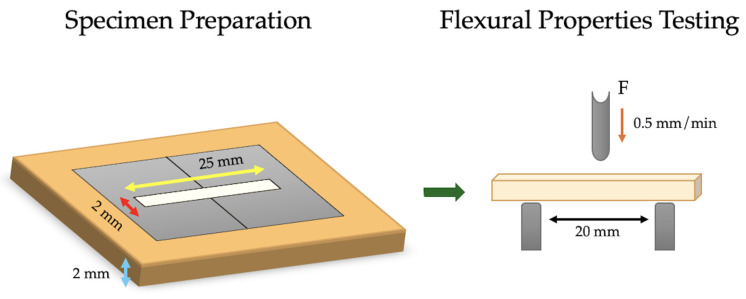
Specimen preparation mold and test parameters for the flexural test in the universal testing machine.

Bar-shaped specimens were created from each IRR (*n* = 6) using a rectangular stainless-steel split mold with a 25 mm × 2 mm × 2 mm central bar shape hollow. All IRRs were mixed, injected into the molds, and light cured according to the manufacturer’s recommendations, just like what was done with the compressive strength testing specimens. All specimens were carefully removed from the molds after disassembling the parts, then stored in distilled water in dark containers at 25 °C for 24 h before testing to allow for complete setting of the materials. All specimens were visually inspected, and defective were discarded and replaced with new specimens. The flexural test specimen mold and testing apparatus are demonstrated in Figure 2.

Each specimen was placed in the universal testing machine on two supports with 20 mm span length to perform the three-point bending test at 1 mm/min crosshead speed. The maximum load at fracture was recorded to calculate flexural strength and modulus. The following formulae were used:

σ_f_ = 3FL/2bh^2^

where f is the maximum load in N; L is the span length in mm; b is the specimen’s width in mm; and h is the specimen’s height in 2 mm.

E_f_ = FL^3^/4wh^3^d

where F is the maximum load in N; L is the span length in mm; w is the specimen’s width in mm; h is the specimen’s height in mm; and d is the deflection at maximum load in mm.

### 2.3. Statistical Analysis

Sample size calculation was performed using the G*Power software (version 3.1.9.6 for Mac OS, Heinrich-Heine-Universität, Düsseldorf, Germany), indicating that a total sample size of 18 specimens per mechanical property resulted in an achieved power of ≈ 80% to detect a large effect difference between the groups at an α = 0.05. The statistical analysis of the results was conducted using R statistical software (Version 4.5.1, R Core Team (2025), Vienna, Austria) and DATAtab statistical calculator (DATAtab: Online Statistics Calculator, DATAtab e.U. Graz, Austria [44]). Quantitative variables were described by calculating the Mean, Standard Deviation (SD), Range (Minimum–Maximum), and 95% confidence interval of the mean.

A Kolmogorov–Smirnov and Levene’s test were run on the mean values of all categorical variables (compressive strength, flexural strength, and flexural modulus) (*p* > 0.05). A two-tailed one-way analysis of variance (ANOVA) at a significance level of *p* < 0.05 was conducted to compare each categorical variable of the three investigated materials, followed by Tukey’s HSD multiple comparison post hoc tests (*p* < 0.05).

## 3. Results

Normal distribution of the mean values of compressive strength (σc), flexural strength (σf), and flexural modulus (Ef) was confirmed using the Kolmogorov–Smirnov test (*p* = 0.33, 0.25, and 0.13, respectively). Levene’s test confirmed the equality of variance between the groups (*p* = 0.18, 0.14, and 0.33, respectively).

**Figure 3:**
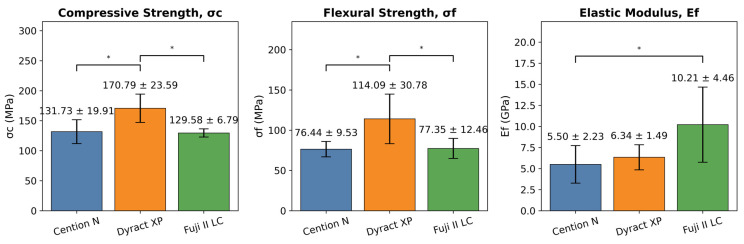
Mean ± standard deviation (SD) of compressive strength (σc), flexural strength (σf), and elastic modulus (Ef) for the three tested ion-releasing restorative materials (IRRs): Cention N, Dyract XP, and Fuji II LC (*n* = 6). Error bars represent SD. * Significant differences among materials at significance (*p* < 0.05) are highlighted.

Dyract XP exhibited the highest compressive and flexural strengths (170.79 ± 23.59 MPa and 114.09 ± 30.78 MPa), whereas Fuji II LC exhibited the highest flexural modulus at 10.21 ± 4.46 GPa. The standard deviation of the σf value in Dyract XP was higher (26.98%) than in the other materials, despite using the standardized specimen preparation protocol in the study. The descriptive statistical data for the categorical variables in the three tested materials Cention N, Dyract XP, and Fuji II LC (*n* = 6) are demonstrated in Figure 3.

The one-way ANOVA indicated significant differences in σc, σf, and Ef between the tested IRRs (*p* < 0.05). Tukey’s HSD post hoc tests showed that Dyract XP had significantly greater mean σc and σf than Cention N and Fuji II LC. However, the mean σc and σf for Cention N and Fuji II LC did not differ significantly. Furthermore, Fuji II LC exhibited a significantly greater mean Ef than Cention N, but was not significantly different than Dyract XP. The complete statistical analysis comparisons with effect sizes are detailed in Table 2.

## 4. Discussion

Dental alkasites, compomers, and resin-modified glass ionomers (RMGICs) represent an important category of ion-releasing restorative materials (IRRs) that serve a vital role in the caries control phase of dental treatment [12,23,35,45]. Their mechanical properties are a key factor in selecting the appropriate restorative material, impacting their clinical performance and durability [23,35,46]. The present study evaluated three important mechanical properties (compressive strength, flexural strength, and flexural modulus) of IRRs: Cention N (an alkasite), Dyract XP (a compomer), and Fuji II LC (an RMGIC). The results revealed significant differences in compressive strength, flexural strength, and flexural modulus between the investigated IRRs (*p* < 0.05), leading to the rejection of the null hypothesis.

The current study utilized the in vitro compressive and flexural tests, following the guidelines outlined in the ISO 4049:2019 specification [47]. The selection of the three-point bending test was used because it reportedly produces results with lower standard deviation and coefficients of variation compared to other test designs, such as the biaxial flexural test [48,49], making it particularly suitable for testing the three investigated IRRs. The investigated alkasite (Cention N) can be either light- or auto-cured, producing specimens with almost the same mechanical properties [40]. Therefore, light curing Cention N in the study was performed according to the manufacturer’s instructions, under the same conditions as the other two investigated IRRs to ensure standardization and reduce variability in the results.

The compressive strength of IRRs is important because the fabricated restoration would be replacing a part of the natural tooth structure and, therefore, should be able to withstand intraoral functional and parafunctional forces [50]. In the current study, Dyract XP exhibited significantly greater values (170.79 ± 23.59 MPa) compared to both Cention N (131.73 ± 19.91 MPa) and Fuji II LC (129.58 ± 6.79 MPa). This superior performance can be attributed to its composition, which combines mechanically strong resin components with the fluoride-releasing capabilities of RMGICs. The improved distribution and type of inorganic fillers (strontium-alumino-sodium-fluoro phosphor-silicate reactive glass fillers) in the Dyract XP, along with its UDMA resin matrix, might have contributed to its greater compressive strength [33]. This could also be associated with an optimized filler-matrix coupling in the material that enhances its polymer network density. This aligns with previous studies that indicated greater compressive strength values of various compomer formulations than RMGICs [32]. Dyract XP’s silanized glass fillers and rapid photo-initiated polymerization may influence the compressive strength by enhancing the cross-linking reaction, resulting in lower residual monomer compared to the slower Cention N’s self-cure reaction [51]. However, a previous study indicated no significant differences in the compressive strength of self-cured versus dual-cured Cention N [40].

It was suggested in the literature that the higher-strength compomer formulations (such as Dyract XP, which was tested in the current study) might have been modified by replacing large quantities of carboxylic acid groups with methacrylates, resulting in a highly cross-linked material that was primarily polymerized through free radical reaction [30,32,33,52]. The significantly lower compressive strength of the alkasite IRR (Cention N) in the study could be related to the manual mixing of the material, which could introduce micro voids and mix variabilities, compared to the industrial manufacturing of the single paste compomer (Dyract XP). This was also suggested in a previous randomized controlled pilot study that tested the marginal integrity of alkasite restorations in endodontically treated molars [53]. The significantly lower compressive strength of Fuji II LC than Dyract XP is supported by the fact that glass ionomers (GICs) generally have lower mechanical properties than resin-based materials [30], which would include the tested polymer (Dyract XP, basically polyacid modified composites).

Even though Cention N had slightly higher compressive strength than Fuji II LC, statistically, they showed comparable values (*p* > 0.05) despite their different material classifications. Cention N is an alkasite composite with a highly crosslinked polymer structure and 58–59 vol% reactive isofillers that reduce polymerization shrinkage forces, relieving internal stresses through matrix-filler silane-mediated chemical bonds [54]. According to a previous study, conventional GICs were found to have lower compressive strength values than alkasites [55]. This can be linked to matrix components leaching through the GIC’s surface porosities, which lowers their compressive strength [56]. Cention N has a higher spherical filler load than GICs, which could also enhance their strength [35], as angled inorganic filler particles usually lead to higher stress concentration within the material [57]. Fuji II LC is basically a GIC that has been enhanced by adding methacrylate resin to enhance the material’s mechanical properties [58], which could explain its comparable compressive strength value with Cention N in the current study.

In this study flexural strength of the IRRs was also compared. This is clinically significant because flexural strength is a key factor in the dental restoration’s resistance to masticatory bending forces and eventual failure [59]. Materials with higher flexural strength are better able to withstand intraoral forces, which improves their durability, functionality, and longevity in the oral environment [48]. According to the ISO standard 4049:2019 for polymer-based restorative materials, the minimum flexural strength for Class 2, Group 1 (i.e., intra-orally light-cured resins) and Class 3 (dual-cure resin-based materials) has to be 80 MPa for Type 1 materials (resin materials claimed by the manufacturer as suitable for occlusal surface restorations) and 50 MPa for Type 2 materials (all other resin materials, including luting) [47,50]. According to the results of the current study, Dyract XP significantly exceeded the requirement (114.09 ± 30.78 MPa), followed by Fuji II LC (77.35 ± 12.46 MPa) and Cention N (76.44 ± 9.53 MPa), which roughly met them. Hiremath et al. found that RMGICs and Cention N had lower flexural strength than the 80 MPa needed for occlusal restorations, making them suitable only for low-stress areas, unlike the results of the current study. This could be attributed to the use of a different RMGIC and light-curing the Cention N, lowering its flexural strength in the current study. It was previously suggested that light curing Cention N would decrease flexural strength because of rapid curing and incomplete bond conversion within the material [30].

In terms of flexural strength, Dyract XP again demonstrated a significantly superior performance (*p* < 0.02). This could indicate that the resin-rich matrix of Dyract XP provided better resistance to fracture under bending forces compared to the other tested materials. This is in accordance with a previous study by Mishra et al., where resin-based composites exhibited greater flexural strengths than alkasites [50]. In the current study, Dyract XP produced greater flexural strength values than those encountered with previous compomer formulations (Dyract AP and Extra) as reported in a study by Bonta et al. [33]. On the other hand, Kaptan et al. reported that bonded auto-cured Cention N had significantly greater flexural strength than other resin-based composites (similar to compomers) [30]. This variability can be due to the different study design, where the specimens in the current study were not bonded to other materials, and the Cention N was light cured. Vaithiyalingam et al. also reported greater flexural strength of Cention N than a light-cured bulk fil resin-based material, which could be attributed to the different mechanical properties of resin materials depending on their constitutional variables [42]. Fuji II LC’s significantly lower flexural strength than Dyract XP in the current study may have been caused by the higher hygroscopic expansion of the material, which could increase its solubility, resulting in a weaker restoration [35]. This result is in accordance with previous literature comparing composites to conventional and modified GICs [32,33].

The higher strength values of Dyract XP in the current study were correlated with a relatively large standard deviation (170.79 ± 23.59 MPa for compressive strength and 114.09 ± 30.78 MPa for flexural strength). The inherent structural properties and failure mechanisms of the Dyract XP compomer, along with potential inconsistencies in filler-matrix coupling and water sorption that can create microscopic regions of weakness, may cause variability and unpredictable failure under stress [60]. Variations in relative humidity during finishing and testing may also affect Dyract XP, which relies on light-cured polymerization and a delayed moisture-dependent acid-base reaction sensitive to environmental factors [61]. Small changes may impact crosslink density and microstructure of specimens. Additionally, Dyract XP may have more plastic deformation with high toughness, shown by its high mean flexural strength and wide range (56.4 to 139.5 MPa). Tougher materials that don’t fail catastrophically often have higher standard deviations, as failure depends on micro-crack propagation and arrest [62,63]. Further investigations into the fracture toughness and behavior of these materials are recommended.

Cention N and Fuji II LC showed no significant difference in flexural strength between them (*p* = 0.997), indicating that, within the limitations of this in vitro study, while alkasite materials represent an advancement in bioactive restoratives, their flexural performance might still be similar to established RMGICs. This is in accordance with previously published literature [48,50]. In contrast, other studies found significantly greater flexural strength of alkasites compared to RMGICs [23,36,64,65]. This might indicate that the investigated alkasite might have inadvertent variability in its resultant strength depending on the mixing, curing, and application procedures. Previous research indicated that changes in the powder-to-liquid ratio, spatulation methods, and hand-mixing techniques can create microvoids and bubbles that weaken the material [66]. This may explain the findings with Cention N in this study. Conversely, RMGIC dispensed in automix capsules, as the case with Fuji II LC used here, maintains an optimal powder/liquid ratio and minimizes air bubble incorporation during mixing, making it easier to handle [66,67,68,69].

The flexural modulus (Ef) results revealed that Fuji II LC exhibited the highest mean flexural modulus (10.21 ± 4.46 GPa), which was significantly greater than that of Cention N (5.5 ± 2.23 GPa; *p* = 0.04). Although the flexural modulus of Dyract XP (6.34 ± 1.49 GPa; *p* = 0.098) was lower than Fuji II LC, it was not statistically significant. Additionally, there was no statistically significant difference between the flexural modulus of Dyract XP and that of Cention N (*p* = 0.879). The higher flexural modulus of Fuji II LC indicates its greater stiffness and brittleness compared to the other IRRs, which may be attributed to RMGIC’s relatively rigid structure, which is partially influenced by the material’s porosity and filler-matrix characteristic [70]. In contrast, Dyract XP and Cention N exhibited lower flexural moduli, indicating they are more flexible, which could be beneficial in clinical scenarios that require some degree of material deformation under load. This is especially relevant in cases of posterior restoration subjected to higher occlusal loads, where a lower flexural modulus would help resist these forces while maintaining the adhesive-tooth bond interface [71]. The absence of a significant difference in this study between Fuji II LC and Dyract XP, despite numerical disparity, may stem from Fuji II LC’s high variability in flexural modulus, indicated by its larger standard deviation and the 73% power achieved in the results of the study to detect flexural modulus differences between the groups. This could result from material heterogeneity, since Fuji II LC is made of a powder and liquid mixed in a capsule, whereas Dyract XP is a pre-fabricated single-paste system. Further studies with larger sample sizes to detect smaller effects with higher power may help clarify these differences.

From a clinical perspective, the material selection process should be based on the specific requirements in each clinical situation. Differences in flexural and compressive strength among Dyract XP (compomer), Cention N (alkasite), and Fuji II LC (RMGIC) directly influence their clinical indications, where higher strength materials would be indicated for stress-bearing areas. Clinical studies have consistently demonstrated a positive correlation between a restorative material’s mechanical properties and a restoration’s fracture resistance and longevity intraorally [53,72,73]. From the results of the current study, Dyract XP’s superior mechanical properties might make it more suitable for the restoration of teeth in high stress-bearing areas as in posterior teeth restorations, while Cention N could possibly offer a balanced combination of adequate strength and bioactive potential. Fuji II LC, while performing inferiorly in the current study to Dyract XP, remains valuable in the restoration of primary and permanent teeth in lower stress-, caries-prone areas due to its previously reported fluoride release and chemical adhesion properties [37].

The strengths of this study lie in its comprehensive comparative analysis of the flexural and compressive strengths among three different ion-releasing restorative materials (Cention N, Dyract XP, and Fuji II LC), providing valuable insights for clinicians in material selection. The utilization of standardized testing methods enhances the reliability of the findings. However, several limitations of this study should be acknowledged. The in vitro design cannot fully replicate the complex oral environment. The current study did not include simulation and aging of the specimens, which could limit the generalizability of the study results. Additionally, the study included a sample size that allowed the detection of a large effect size but might have missed the detection of smaller effect sizes. Therefore, future research should investigate the long-term durability of these materials under conditions that better simulate the oral cavity, including thermocycling and mechanical fatigue testing. Additionally, the study focused on specific mechanical properties of the materials without considering others, such as wear resistance, thermal stability, color stability, and mechanical stability. Therefore, future studies comparing other mechanical properties, long-term durability, and the materials’ bioactivity, ion-release with immersion testing, and caries-inhibiting potential are recommended as they would provide a more comprehensive understanding of their clinical performance and enhance the generalizability of the results. Additionally, the limited sample size (*n* = 6 per material), with the moderate achieved power in the flexural modulus comparison (73%), may not have captured the full variability of the IRRs. The study also showed a high standard deviation for Dyract XP’s strength that could limit results generalizability. Furthermore, further research, particularly in clinical settings, is warranted to better understand the long-term performance and suitability of these materials in restorative dentistry.

## 5. Conclusions

Based on the study results, it can be concluded that Dyract XP compomer (compressive strength: 170.79 ± 23.59 MPa and flexural strength: 114.09 ± 30.78 MPa) represents a strong filling material that would be suitable for the restoration of both anterior and posterior teeth. The alkasite Cention N (compressive strength: 131.73 ± 19.91 MPa and flexural strength: 76.44 ± 9.53 MPa) and the Fuji II LC RMGIC (compressive strength: 129.58 ± 6.79 MPa and flexural strength: 77.35 ± 12.46 MPa) presented similar strength values, while Fuji II LC presented as the material with the highest flexural modulus (10.21 ± 4.46 GPa), which could limit its applicability as a posterior restorative material compared to Cention N. Future studies are recommended to assess the long-term clinical performance of these ion-releasing restorative materials under simulated oral conditions, including thermocycling and mechanical fatigue testing, while evaluating other mechanical properties and bioactivity. This emphasizes the continuing importance of developing innovative solutions in restorative dentistry that adhere to the principles of minimal intervention and improved dental health.

## Figures and Tables

**Table 1:** Tested materials’ type, composition, and manufacturer information.

Name	Material	Manufacturer	Composition
Cention N	Alkasite	Ivoclar Vivadent, Schaan, Liechtenstein	Powder: 25–35% Calcium fluorosilicate glass (alkaline), 20–30% Barium-aluminum silicate glass, 10–20% fluorosilicate glass, Barium glass, 5–10% Ytterbium trifluoride, 15–25% isofiller (Copolymer), <1% initiator (self-cure initiator: copper salt & thiocarbamide, and light cure initiator: ivocerin and acyl phosphine oxidephotoinitiator), <0.1 Pigment. Liquid: 95–97% Urethane dimethacrylate (UDMA), Tricyclodecan-dimethanoldimethacrylate (DCP), Tetramethylxylylen-diurethanedimethacrylate (aromatic-aliphatic UDMA), and Polyethylene glycol 400 dimethacrylate (PEG-400 DMA). 1–2% additives, 2–3% self-cure initiator (hydroperoxide), and <1% stabilizer. Particle size: 0.15–0.17 μm. (58–59 vol% inorganic fillers)
Dyract XP	Compomer	Dentsply Sirona, Konstanz, Germany	UDMA, carboxylic acid modified dimethacrylate, TEGDMA, trimethacrylate resin (TMPTMA), dimethacrylate resins, camphorquinone, ethyl-4 (dimethylamino) benzoate, butylated hydroxy toluene (BHT), strontium-alumino-sodium-fluoro phosphor-silicate glass, highly dispersed silicon dioxide, strontium fluoride, iron oxide pigments and titanium oxide pigments. Particle size: 0.8 μm. (50 vol% inorganic fillers).
Fuji II LC	Resin Modified Glass-ionomer	GC Europe N.V, Leuven, Belgium	Powder: 100% fluoroaluminosilicate glass (FAS) Liquid: 35% 2-Hydroxyethyl methacrylate (HEMA), 25% distilled water, 24% polyacrylic acid, 6% tartaric acid and 0.10% camphorquinone (photoinitiator), bisphenol A-glycidyl methacrylate (Bis-GMA), and traces of triethylene glycol dimethacrylate (TEGDMA). Particle size: 5.9 μm. (47.0 vol% inorganic fillers)

**Table 2:** The one-way ANOVA and Tukey’s HSD pairwise comparisons of the compressive strength, flexural strength, and flexural modulus of the investigated materials.

Compressive Strength (σ_c_)							
ANOVA	Sum of Squares	df	Mean Square	F	η^2^	Cohens f^2^	*p*
Material	6457.68	2	3228.84	9.69	0.56	1.14	0.002 *
Residual	4995.89	15	333.06			(Power = 97.9%)	
Total	11,453.57	17					
Tukey’s HSD	Mean difference		95% CI lower limit	95% CI upper limit			*p*
Cention N-Dyract XP	39.06		11.69	66.43			0.006 *
Cention N-Fuji II LC	2.15		−25.22	29.52			0.977
Dyract XP-Fuji II LC	41.21		13.84	68.58			0.004 *
**Flexural Strength (** **σ_f_)**							
ANOVA	Sum of Squares	df	Mean Square	F	η^2^	Cohens f^2^	*p*
Material	5535.03	2	2767.52	6.96	0.48	0.96	0.007 *
Residual	5966.76	15	397.78		(large)	(Power = 91.9%)	
Total	11,501.8	17					
Tukey’s HSD	Mean difference		95% CI lower limit	95% CI upper limit			*p*
Cention N-Dyract XP	37.64		7.73	67.55			0.013 *
Cention N-Fuji II LC	0.91		−29	30.82			0.997
Dyract XP-Fuji II LC	36.74		6.83	66.65			0.016 *
**Flexural modulus (E_f_)**							
**Analysis of variance**	Sum of Squares	df	Mean Square	F	η^2^	Cohens f^2^	*p*
Material	75.82	2	37.91	4.21	0.36	0.75	0.035 *
Residual	135.21	15	9.01		(large)	(Power = 73%)	
Total	211.02	17					
Tukey’s HSD	Mean difference		95% CI lower limit	95% CI upper limit			*p*
Cention N-Dyract XP	0.84		−3.66	5.35			0.879
Cention N-Fuji II LC	4.71		0.21	9.22			0.04 *
Dyract XP-Fuji II LC	3.87		−0.63	8.37			0.098

Where * indicates statistically significant differences at *p* < 0.05.

## Data Availability

All study data are supplied in the manuscript.

## Abbreviations

The following abbreviations are used in this manuscript:

ANOVA	Analysis of variance
Bis-GMA	Bisphenol A-glycidyl methacrylate
CI	Confidence interval
DCP	Tricyclodecan-dimethanoldimethacrylate
DMA	Dimethacrylate
FAS	Fluoroaluminosilicate glass
GIC	Glass ionomer cement
HEMA	2-Hydroxyethyl methacrylate
LED	Light emitting diode
RMGIC	Resin-modified glass ionomer cement
TEGDMA	Triethylene glycol dimethacrylate
TMPTMA	Trimethacrylate resin
UDMA	Urethane dimethacrylate
